# Effects of Acute Vitamin C plus Vitamin E Supplementation on Exercise-Induced Muscle Damage in Runners: A Double-Blind Randomized Controlled Trial

**DOI:** 10.3390/nu14214635

**Published:** 2022-11-03

**Authors:** María Martínez-Ferrán, Víctor Cuadrado-Peñafiel, Juan Manuel Sánchez-Andreo, Marta Villar-Lucas, Mónica Castellanos-Montealegre, Agustín Rubio-Martín, Carlos Romero-Morales, Soraya Casla-Barrio, Helios Pareja-Galeano

**Affiliations:** 1 Faculty of Sports Sciences, Universidad Europea de Madrid, 28670 Madrid, Spain; 2 Faculty of Health Sciences, Universidad Isabel I, 09003 Burgos, Spain; 3 Department of Physical Education, Sport and Human Movement, Universidad Autónoma de Madrid, 28049 Madrid, Spain; 4 Hospital 12 de Octubre, 28041 Madrid, Spain; 5 Faculty of Sports Sciences, Universidad de Castilla-La Mancha, 45002 Toledo, Spain; 6 Tigers Running Club, 28009 Madrid, Spain; 7 Faculty of Nursing, Universidad de Comillas, 28015 Madrid, Spain

**Keywords:** antioxidants, ascorbic acid, tocopherols, athletic performance, muscle damage

## Abstract

Considering the existing controversy over the possible role of acute antioxidant vitamins in reducing exercise-induced muscle damage (EIMD), this doubled-blind, randomized and controlled trial aimed to determine whether supplementation with vitamins C and E could mitigate the EIMD in endurance-trained runners (*n* = 18). The exercise protocol involved a warm-up followed by 6 to 8 bouts of 1 km running at 75% maximum heart rate (HRmax). Two hours before the exercise protocol, participants took the supplementation with vitamins or placebo, and immediately afterwards, blood lactate, rate of perceived exertion and performance were assessed. At 24 h post-exercise, CK, delayed onset muscle soreness and performance were determined (countermovement jump, squat jump and stiffness test). The elastic index and vertical stiffness were calculated using a stiffness test. Immediately after the exercise protocol, all participants showed improved maximum countermovement jump, which only persisted after 24 h in the vitamin group (*p* < 0.05). In both groups, squat jump height was significantly greater (*p* < 0.05) immediately after exercise and returned to baseline values after 24 h. The elastic index increased in the vitamin group (*p* < 0.05), but not in the placebo group. In both groups, lactate levels increased from pre- to immediately post-exercise (*p* < 0.05), and CK increased from pre- to 24 h post-exercise (*p* < 0.05). No significant differences between groups were observed in any of the variables (*p* > 0.05). Vitamin C and E supplementation does not seem to help with EIMD in endurance-trained individuals.

## 1. Introduction

Exercise-induced muscle damage (EIMD) is a common response to a prolonged or high-intensity bout of exercise, occurring in a transient manner and as part of muscle repair and adaptation [1,2]. This type of response can temporarily diminish muscle function and also cause muscle soreness, swelling and raised blood levels of intramuscular proteins [3].

Interest in post-exercise recovery has drastically increased in the last few decades [4], and nutrition is a key strategy in this process [5,6]. Several dietary and supplementation strategies have been proposed to attenuate symptoms of EIMD [1,7,8]. One of the strategies tested is based on antioxidant supplementation, which targets reactive oxygen and nitrogen species (RONS) as factors involved in EIMD [2,3,9].

Several studies have examined whether vitamin C (VitC) and vitamin E (VitE), either alone or in combination, are able to mitigate EIMD [10]. However, while some data point to a muscle protective effect of these antioxidant vitamins, the evidence so far is inconclusive and not all studies have shown their clear benefits [10,11]. In effect, recognized institutions such as the Australian Institute of Sport have identified a need for more research regarding the effects of these vitamins [12,13].

One of the mechanisms of action of antioxidants against EIMD is thought to be limiting lipid peroxidation [10,11], which leads to sarcolemma disruption [14]. VitE is the most abundant fat-soluble antioxidant that protects against lipid peroxidation [15,16], whereas VitC performs multiple antioxidant actions, owing to its capacity to react with several RONS and consequently decrease lipid peroxidation. Additionally, both vitamins interact with each other to recycle VitE, which is regenerated by VitC [17].

Besides the possible benefits of acute supplementation with antioxidants, there is growing literature suggesting that RONS generated during exercise have an essential role in regulating cell signaling pathways and human redox-sensitive transcription factors [18]. RONS mediate training adaptations such as mitochondrial biogenesis, skeletal muscle hypertrophy or induction of the endogenous antioxidant system [19,20]. Consequently, supplementation with high doses of antioxidants could have the effect of blunting certain adaptations to exercise training [21,22,23].

Factors such as the type and period of supplementation, or modality and duration of exercise could influence the effect that antioxidants have on the response and adaptations to exercise. Accordingly, most beneficial strategies have involved acute supplementation and fatiguing-type exercise [24]. Hence, it could be useful to consider acute antioxidant supplementation to enhance performance in exercise modalities for which adaptive responses are irrelevant. However, this issue remains unclear as evidence is still scarce [17]. Based on the results of our previous systematic reviews [5,6], we hypothesized that this supplementation could have a protective effect on EIMD and would not benefit physical performance.

Taking into account the wide use of antioxidant vitamins [25] and the existing controversy over their possible role in minimizing EIMD, this double-blind randomized controlled trial (RCT) investigated whether acute VitC/VitE supplementation could mitigate EIMD and improve performance in endurance-trained runners.

## 2. Materials and Methods

The study design was double-blind, RCT, registered at Clinical Trials (https://clinicaltrials.gov) (accessed on 15 June 2021) with the identifier NCT05127928.

### 2.1. Participants

Subjects were invited to participate on a voluntary basis if they were healthy and non-smokers (February 2022). The participants recruited were 18 recreationally endurance-trained male runners aged 39 to 58 years, belonging to a Running Club in Madrid. Subjects performed 4 to 5 days of training per week (40–60 km/week), and they had between 3 to 8 years of experience in endurance training. Only men were selected to be able to compare our results with previous literature, as most of the research in this area has been conducted on the male population [5,6]. Participants were excluded if they had cardiometabolic or musculoskeletal disorders, were smokers, or used any type of supplementation. The intake of dietary supplements was not allowed from two weeks prior to the intervention until it ended.

Before initiating the study, subjects were informed of the study procedures, goals and possible risks, and written consent was obtained. This study was approved by the Research Ethics Committee of the Universidad Europea (protocol code: CIPI/20/209) and conducted in accordance with the principles of the Declaration of Helsinki.

### 2.2. Experimental Design

Participants were randomly allocated to the VitC plus VitE (VIT) group or the placebo (PLA) group, in which supplementation was taken 2 h prior to an exercise protocol. Physical exercise was avoided during the 48 h before the intervention.

Randomization was based on a computer-generated random allocation sequence and was conducted by an external investigator. No researcher participating in the study had access to it. During the trial, participants and personnel evaluating outcomes were blinded to group allocation.

First, a Cooper test was conducted to estimate maximal oxygen consumption (VO_2max_). Participants covered the furthest distance possible in 12 min following the traditional protocol [26]. The total distance completed and heart rate (HR) during the protocol were measured. VO_2max_ was calculated using the following equation: 22.351 × distance covered (km)—11.288 [27].

Participants fasted overnight, arriving in the morning when blood and lactate samples were collected and performance was assessed. Next, subjects ingested the supplement and ate a standardized breakfast (4.7 kcal/kg, 1 g/kg carbohydrate, 0.1 g/kg protein) based on bread and jam or honey and water. The exercise protocol commenced with a warm-up based on 15 min of running at an intensity of 60% of maximum HR (HR_max_). After 2 min of rest, participants executed 6 to 8 bouts of 1 km running at 75% of HR_max_ separated by 1 min of recovery. If HR was less than 75% HR_max_, they did not perform bouts 7 and 8. Finally, subjects completed 10 min of running at 70% HR_max_. Exercise intensity was controlled by HR in order to produce a physiological response to effort without generating cell damage. Therefore, by verifying that HR did not return to baseline, we were able to estimate that the intensity exceeded the predicted physical capacity of each participant.

Immediately after the exercise session, lactate samples were collected and performance was measured. Participants also reported their rate of perceived exertion (RPE). After finishing the tests, participants consumed a recovery meal based on 1.2 g/kg of carbohydrate and 0.2 g/kg of protein. Twenty-four hours later, subjects were subjected again to blood tests and performance measurement. They also described their delayed onset muscle soreness (DOMS). The trial was conducted in March 2022 in Madrid.

### 2.3. Supplementation with Antioxidant Vitamins

VitE capsules contained 235 mg of DL-α-tocopherol acetate, VitC capsules contained 1000 mg of ascorbic acid, and PLA capsules contained maltodextrin (Vecos Nucoceutical). The PLA capsules without VitC or VitE were indistinguishable in terms of shape, appearance and taste to the vitamin pills. Capsules were provided in individual bags identified with a participant code. Labeling was completed by an investigator blinded to identification codes, and allocation was performed by a blinded investigator.

### 2.4. Diet Control and Body Composition Anaylsis

Every participant followed a diet adapted to their body weight constructed by a dietitian, according to nutrition guidelines for endurance sport [28,29], starting two days before the intervention and continuing until the end of all tests. The diet was adjusted to provide 6 g/kg/d two days before the intervention and 7 g/kg/d of carbohydrate from the day before trial, until the end of the trial. Protein intake was adjusted to 1.6 g/kg and distributed in three main meals and two collations [8,28,29]. Foods with high amounts of antioxidants, such as more than four cups of tea or coffee, more than two fruit juices and alcoholic beverages were avoided two days prior to the intervention. During the intervention, coffee and tea were also avoided.

In order to preserve hydration state, participants were asked to maintain their urine color between level 1 and 3 using The Urine Color Chart (Human Kinetics, Champaign, IL, USA), mainly before the intervention.

Regarding body composition data, body mass (kg), body fat (%), muscle mass (kg), bone mass (kg) and visceral adipose tissue were determined by a Tanita InnerScan V BC-601 device the intervention day prior to the blood analysis.

### 2.5. Outcome Measures

The primary outcomes were the differences between the VIT and PLA groups in muscle damage and performance. Secondary outcomes included the differences between groups in lactate, DOMS and RPE.

#### 2.5.1. Muscle Damage

Venous blood for CK determination was collected from a superficial vein in the antecubital fossa into standard Vacutainer^®^ tubes containing K3EDTA. Tubes were left upright for 45 min to allow for complete blood clotting before centrifugation. Serum was then separated by centrifugation at 3500× *g* rpm for 15 min at room temperature, stored in aliquots and kept frozen at −80 °C until analysis. Creatine kinase was determined in a Roche Cobas^®^ c 311 apparatus (Roche Diagnostics GmbH, Penzberg, Germany) according to the manufacturer’s specifications. CK was determined 24 h after exercise [30].

#### 2.5.2. Lactate

Lactate concentrations were measured in a capillary blood sample obtained from participants’ fingertips using a portable analyzer (Lactate 2, Arkray, Kyoto, Japan) [31,32].

#### 2.5.3. Physical Performance

Participants performed three different jumps: countermovement jump (CMJ), squat jump (SJ) and stiffness test (ST). For the CMJ, subjects started from an upright position with hands on waist and then executed a countermovement jump by flexing the knees to 90° and jumping as high as possible. During the flight stage, they were instructed to keep their knees extended to 180°, without hyperextending the hips [33]. Jump height was measured on an infrared platform (Optojump, Microgate, Bolzano, Italy).

Immediately after the CMJ, participants performed a SJ, starting from a standing position and crouched to 90° knee flexion, followed immediately by a jump to maximum height [34]. Jump height was recorded using the infrared platform. The best and worst of five CMJ and SJ jump heights were eliminated and the mean of the three remaining attempts were used in the data analysis.

Finally, subjects undertook a ST consisting of a countermovement jump followed by seven jumps with knees extended, landing after the seventh jump and controlling the vertical position without doing further hops [35]. Neuromuscular performance was determined from these jumps, in terms of jump height, elastic index (EI%) and vertical stiffness (Kvert).

#### 2.5.4. DOMS and RPE

DOMS was determined using a 10-point visual analogue scale from “0”, indicating total absence of pain, to “10”, indicating the maximum level of tolerable pain [36]. This soreness rating was used for the upper body (above the hip), upper part of the legs (from the hip to the knees) and lower part of the legs (from the knees to the ankles). RPE was assessed with a 10-point scale [37] adapted by Foster et al. [38].

### 2.6. Statistical Analysis

The normality of the distribution of the variables was confirmed with the Shapiro–Wilk test. Baseline differences were analyzed with Student’s *t*-test and relative changes within each group were assessed using a paired Student’s *t* test or two-way ANOVA with Bonferroni-adjusted post hoc tests. Between-group differences were detected by two-way ANOVA and ANCOVAS, adjusting values to baseline assessments. Results are presented as the mean (and standard deviation), effect size, *p*-value and observed power. For inter-group comparisons, 95% confidence interval (CI) and mean differences were determined. The effect size was determined by Cohen’s d for Student’s *t* test (magnitude: 0 to 0.2 = trivial, 0.2 to 0.6 = small, 0.6 to 1.2 = moderate, 1.2 to 2 = large, and >2 = very large [39]), by Wilcoxon’s effect size for Wilcoxon test (magnitude: small effect = 0.10–0.3, moderate = 0.30–0.5, large ≥ 0.5 [40]) or by Eta squared (η^2^) for two-way ANOVA (magnitude of effect size: 0.01 = small, medium = 0.06, large = 0.14 [40]). For variables not showing a normal distribution, nonparametric tests were used. All data were analyzed with the Statistical Package for the Social Sciences version 23 (IBM, Chicago, IL, USA). Significance was set at *p* < 0.05.

## 3. Results

### 3.1. Participant Characteristics

**Figure 1:**
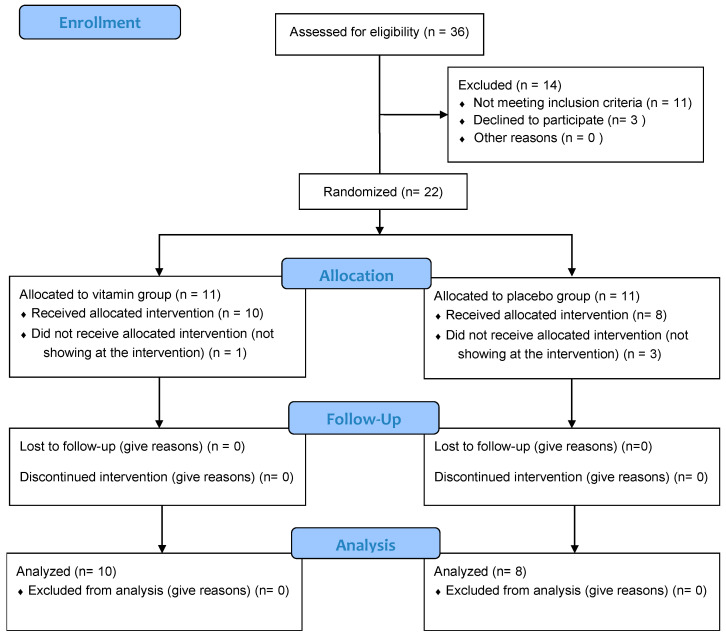
CONSORT (Consolidated Standards of Reporting Trials) Flow Diagram.

Participant study flow is presented according to the Consolidated Standards of Reporting Trial (CONSORT) diagram (Figure 1). Of the eleven subjects randomly assigned to each group, ten received vitamin supplementation and eight PLA. Four participants did not show up on the day of the intervention. Participant characteristics are provided in Table 1. No significant differences between groups were observed (Table 1).

### 3.2. Muscle Damage

Twenty-four hours after the exercise protocol, CK levels were significantly higher than at pre-exercise in both groups (*p* < 0.05) (Table 2) and there were no between-group differences (Table 3).

### 3.3. Lactate Levels

In both groups, blood lactate levels rose significantly from pre-exercise to immediately after exercise (*p* < 0.05) (Table 2). No significant between-group differences were observed (Table 3).

### 3.4. Performance

Immediately after the exercise protocol, maximum CMJ height was significantly higher than before exercise in both groups (*p* < 0.001). After 24 h, this height remained elevated only in the VIT group (*p* < 0.05), while in the PLA group it fell to baseline levels (*p* > 0.05). In both groups, maximum SJ height was significantly greater (*p* < 0.05) immediately after exercise and returned to baseline values after 24 h (*p* > 0.05). A significant increase in EI% was produced from pre- to 24 h post-exercise in the VIT group (*p* < 0.05) (Table 2).

Inter-group comparisons (time × group) revealed no significant difference in performance (*p* > 0.05) (Table 3).

### 3.5. DOMS and RPE

No significant differences in RPE and DOMS were detected between the groups (*p* > 0.05) (Table 4).

## 4. Discussion

This study was designed to examine whether acute supplementation with VitC plus VitE could mitigate EIMD and improve athletic performance in endurance-trained individuals. To date, several investigations have explored the effects of chronic supplementation with VitC and VitE on EIMD [10]. However, acute supplementation protocols have only tested the intake of VitC [41,42,43] or VitE [44]. Therefore, as far as we know, this is the first investigation to address the effects of acute supplementation with both VitC and VitE before an exercise session.

Two studies conducted in young untrained subjects have assessed muscle damage measured through CK following acute supplementation with VitC or VitE. No significant differences were detected between individuals taking VitC (1000 mg) compared to PLA after running 30 min at 75% VO_2max_. In both these studies, it was observed that CK only remained elevated for 24 h after exercise in the PLA group [41,42]. Another two studies in physically-active men examined the effects of an antioxidant supplementation on the response to a 90 min high-intensity interval training session [45,46]. In the first study [45], participants received 1 g of VitC 2 h before exercise, and in the second one [46] participants took 200 mg of VitC twice a day during the 3 days after exercise. In both studies, CK levels increased significantly after exercise, and no differences emerged between groups. Therefore, the results of all of these articles are in line with our results showing that supplementation with VitC and VitE have no effect on CK.

In contrast, 800 mg of VitC supplemented 3 h before exercise and 21 h after exercise led to a significant reduction in CK concentrations 24 h after eccentric exercises in young basketball players [43]. Beneficial effects of VitE supplementation in response to exercise in conditions of hypoxia compared to normoxia have also been reported [44]. Thus, acute supplementation might only be effective in certain conditions, such as in exercise under hypoxia [44] or eccentric exercise [43].

Several groups have assessed the effects of chronic VitC and VitE supplementation before an acute exercise session in runners [47,48,49]. In these studies, no effects on CK levels were found when VitC (500–1000 mg/d) plus VitE (300–1000 IU/d) were taken for 2–6 weeks before a 50 km ultramarathon, a marathon, and a 1.5 h downhill exercise, respectively. Rokitzki et al. [50] reported that supplementation with VitC (200 mg/d) and VitE (400 IU/day) for 4.5 weeks before the marathon gave rise to reduced CK concentrations 24 h after exercise. However, the authors miscalculated the *p*-value, thus, the difference was really not significant, as observed in the previous studies and our investigation.

In the study by Cannon et al. [51], the effects of VitE supplementation with 800 IU/d over 48 days were assessed in younger and older sedentary men after performing 45 min of downhill running. It was revealed that in the older SUP group, CK was significantly higher before exercise and 2 days after, than in the older PLA group. In comparison to the younger PLA group, older participants given PLA showed significantly reduced levels of CK. Peters et al. [52] reported that supplementation with VitC for 7 days prior to a 90 km running event, as well as on the event day and two days later, in runners exacerbated the CK and C-reactive protein response to exercise.

On the contrary, Itoh et al. [53] found that supplementation with VitE (250 mg) for 4 weeks before and during a 6-day running training protocol led to significantly reduced concentrations of CK and lactate dehydrogenase 24 h after exercise in trained male runners.

The significant increase of CK in this study, in response to exercise in all participants with no differences between our SUP and PLA groups, is in line with most data reported in runners receiving VitC and/or VitE supplements [47,48,49,50]. It should be highlighted that participants of all studies detecting beneficial effects on CK of acute or chronic VitC or VitE supplementation were young, i.e., under 25 years of age [10].

EIMD can have different consequences, such as DOMS [11]. Different theories have been proposed to explain the mechanisms underlying DOMS; for instance, there is an increased release of RONS in response to exercise [10]. However, most authors have found that antioxidant supplementation does not diminish muscle soreness [11,54], as we observed in our investigation. Hence, we would argue that supplementation with VitC/VitE combined in athletes aged over 30 years does not reduce EIMD, as measured through blood CK levels, and neither does it affect perceived DOMS.

While RONS are thought to play a role in modulating cell signaling pathways and controlling numerous redox-sensitive transcription factors, it has been well established that increased RONS production promotes skeletal muscle contractile dysfunction resulting in muscle fatigue [55]. Consequently, a common action attributed to antioxidant vitamin supplementation is increased performance, yet the available literature suggests that chronic VitE and/or VitC supplementation does not improve endurance performance [10], as our study points out following an acute supplementation protocol. Despite this, running blocks at speeds below maximum aerobic speed (MAS), has been found to lead to post-activation potentiation (PAP) (in both SUP and PLA groups), producing greater activation of fast twitch fibers [56,57] and causing a significant increase in post-exercise jumping capacity. Here, after 24 h, only the VIT group participants recovered the ability to apply force influenced by improved muscle-tendon performance (due to a better use of elastic energy). We also observed an increase (non-significant) in vertical stiffness in both groups immediately after exercise. Running blocks at increasing speed (without reaching MAS) has been reported to increase Kvert [56], and this increase persists when MAS is reached [58]. In our study, running speed was maintained at an intensity close to the anaerobic threshold, and for this reason, there was a tendency for Kvert to increase, but not significantly. The coincidence of both fatigue and neuromuscular activation in middle- and long-distance runners warrants further investigation.

As far as we know, the literature continues to lack data regarding the effects of acute supplementation with VitC and VitE on exercise performance in humans [10,17,24,59]. Additionally, several investigations have confirmed our finding that VitC and/or VitE supplementation has no effect on the blood lactate response to exercise [45,46,60,61,62].

### Limitations and Strength

A key strength of this investigation is its experimental design consisting of a double-blind RCT. Some studies have failed to estimate food intake and others have used food recall questionnaires which are open to biased responses. In our investigation, participants were given a personalized diet to follow based on nutritional recommendations for endurance training and avoiding significant sources of dietary antioxidants. An important limitation of this investigation is the limited simple size (N = 18) compared to other studies with the similar objectives and sample sizes from 19 to 32 [36,48,49,51,63,64,65,66]. Moreover, the power analysis of the inter-group differences is low (<0.6). Finally, training intensity was based on HR rather than a direct method of measuring VO_2max_.

## 5. Conclusions

VitC plus VitE supplementation did not attenuate EIMD in trained subjects aged over 30 years. Additionally, this antioxidant supplementation had no effects on perceived DOMS and performance. Accordingly, acute supplementation with antioxidant vitamins does not appear to produce any beneficial or detrimental effect on EIMD, DOMS or endurance athletes.

## Figures and Tables

**Table 1:** Participant characteristics.

	Group VIT	Group PLA	p-Value
Age (years)	47.90 (5.75)	46.76 (4.60)	0.656 ^a^
VO_2max_ (mL/min)	53.64 (11.77)	49.65 (9.27)	0.460 ^a^
HRmax (BPM)	174.75 (4.15)	174.53 (4.00)	0.655 ^a^
Body mass (kg)	74.84 (10.94)	75.00 (9.82)	1.00 ^b^
Body fat (%)	21.81 (5.37)	19.31 (4.32)	0.302 ^a^
Muscle mass (kg)	55.22 (5.60)	57.40 (7.10)	0.476 ^a^
Bone mass (kg)	2.90 (0.28)	3.025 (0.35)	0.416 ^a^
Total body water	55.58 (4.04)	57.64 (3.34)	0.264 ^a^
Visceral adipose tissue	9.00 (3.20)	8.25 (1.98)	0.571 ^a^

All values show the mean (standard deviation); Inter-group differences were calculated by independent *t*-test ^a^ and Mann–Whitney U test ^b^. Body composition was determined with a Tanita InnerScan V BC-601 device. Abbreviations: BPM (beats per minute); PLA (placebo supplementation); HR_max_ (maximum heart rate); VIT (vitamin C plus vitamin E supplementation); VO_2max_ (maximum oxygen consumption).

**Table 2:** Performance, muscle damage and blood lactate data recorded at pre-exercise, immediately after exercise, and 24 h after exercise and intra-group differences.

	Group	PRE	POST	24 h	Effect Size	*p*–Value (Intragroup × Time)	Observed Power
CMJ	VIT	22.63 (5.02)	26.71 (6.27) *	25.41 (6.54) *	0.696	0.000 ^a^	1.000
	PLA	20.97 (4.21)	25.29 (5.32) *	21.89 (5.02) **	0.750	0.000 ^a^	1.000
SJ	VIT	21.65 (4.67)	24.74 (6.10) *	22.78 (5.73) **	0.603	0.000 ^a^	0.994
	PLA	20.29 (4.38)	22.91 (5.32) *	20.21 (4.31) **	0.656	0.001 ^a^	0.989
EI (%)	VIT	4.48 (4.01)	8.25 (4.66)	11.52 (5.13) *	0.400	0.010 ^a^	0.820
	PLA	4.16 (9.19)	11.25 (9.52)	8.02 (5.85)	0.208	0.195 ^a^	0.319
Kvert	VIT	159.65 (63.14)	173.45 (56.72)	173.92 (57.68)	0.068	0.533 ^a^	0.142
	PLA	161.51 (28.28)	177.86 (56.25)	157.27 (38.88)	0.152	0.315 ^a^	0.228
Lactate	VIT	1.64 (0.40)	6.02 (2.25)	-	−1.006 ^y^	0.000 ^b^	-
	PLA	1.86 (1.01)	5.73 (2.36)	-	−0.891 ^yy^	0.012 ^c^	-
CK	VIT	103.03 (15.45)	-	302.40 (66.76)	−0.886 ^yy^	0.005 ^c^	-
	PLA	125.06 (65.64)	-	233.86 (47.48)	−2.493 ^y^	0.000 ^b^	-

Intra-group differences were calculated by two-way ANOVA with Bonferroni-adjusted post hoc tests ^a^, paired sample *t*-test ^b^ and Wilcoxon test ^c^. Effect size was determined by Eta squared (η^2^), Cohen’s d ^y^ or Wilcoxon effect size ^yy^. * Significant difference versus baseline (*p* < 0.05). ** Significant difference versus immediately post-exercise (*p* < 0.05). Abbreviations: CK (creatine kinase); CMJ (countermovement jump); EI% (elastic index); K_vert_ (vertical stiffness); PLA (placebo supplementation); SJ (squat jump); VIT (vitamin C plus vitamin E supplementation).

**Table 3:** Performance, muscle damage and lactate data recorded at pre-exercise, immediately after exercise, and 24 h after exercise and inter-group differences.

	Mean Differences	Effect Size (η^2^)	95% CI	*p*-Value (Intergroup × Time)	Observed Power
CMJ	2.20	0.007	(−3.23, 7.63)	0.072 ^a^	0.522
SJ	1.92	0.002	(−3.18, 7.02)	0.377 ^a^	0.196
IE (%)	0.27	0.038	(−4.09, 4.63)	0.282 ^a^	0.264
KVERT	3.46	0.009	(−43.44, 50.36)	0.526 ^a^	0.150
CK	90.27	0.077	(−71.07, 251.60)	0.252 ^b^	0.201
Lactate	0.13	0.009	(−2.25, 2.52)	0.907 ^b^	0.051

Between-group differences were detected by two-way ANOVA ^a^ and ANCOVAS ^b^. Effect size was determined by Eta Squared (η^2^). CI (confidence interval); CK (creatine kinase); CMJ (countermovement jump); EI % (elastic index); K_vert_ (vertical stiffness); PLA (placebo supplementation); SJ (squat jump); VIT (vitamin C plus vitamin E supplementation).

**Table 4:** Delayed-onset muscle soreness, rate of perceived exertion and intergroup differences.

	Group VIT	Group PLA	*p*-Value (Intergroup) ^a^
RPE	5.30 (2.71)	5.00 (2.00)	0.829
DOMS 24 h upper body	1.60 (0.84)	2.25 (1.91)	0.762
DOMS 24 h upper legs	2.90 (2.18)	3.88 (2.30)	0.408
DOMS 24 h lower legs	2.50 (2.22)	4.00 (3.02)	0.408

All values show the mean (standard deviation); ^a^ Mann–Whitney U test was used to detect between group differences. Abbreviations: DOMS (delayed-onset muscle soreness); RPE (rate of perceived exertion).

## Data Availability

The data presented in this study are available upon reasonable request from the corresponding author.
